# Disease Activity Indices in Rheumatoid Arthritis: Comparative Performance to Detect Changes in Function, IL-6 Levels, and Radiographic Progression

**DOI:** 10.3389/fmed.2021.669688

**Published:** 2021-05-31

**Authors:** Sebastián C. Rodriguez-García, Nuria Montes, José Ivorra-Cortes, Ana Triguero-Martinez, Luis Rodriguez-Rodriguez, Isabel Castrejón, Loreto Carmona, Isidoro González-Álvaro

**Affiliations:** ^1^Rheumatology Department, Hospital Universitario La Princesa, Instituto de Investigación Sanitaria - Instituto Princesa (IIS-IP), Madrid, Spain; ^2^Rheumatology Department, Hospital Universitario y Politécnico La Fé, Valencia, Spain; ^3^Rheumatology Department, Hospital Clínico San Carlos, Instituto de Investigación Sanitaria San Carlos (IdiSSC), Madrid, Spain; ^4^Rheumatology Department, Hospital General Universitario Gregorio Marañón, Madrid, Spain; ^5^Instituto de Investigación Músculo-Esquelética (InMusc), Madrid, Spain

**Keywords:** rheumatoid arthritis, outcome assessment (health care), statistical analysis, interleukin-6, radiographic progression, disease activity score

## Abstract

**Objective:** To compare the capacity of various disease activity indices to evaluate changes in function, IL-6 levels, and radiographic progression in early and established rheumatoid arthritis (RA).

**Methods:** Secondary data analysis of a clinical trial assessing the efficacy of tocilizumab in patients with established RA (ACT-RAY) and a longitudinal prospective register of early arthritis (PEARL). Targeted outcomes were changes in physical function, measured with the health assessment questionnaire (HAQ), IL-6 serum levels, and radiographic progression. The “Hospital Universitario La Princesa Index” (HUPI), DAS28 using erythrocyte sedimentation rate and SDAI were the disease activity indices compared. Models adjusted for age and sex were fitted for each outcome and index and ranked based on the *R*^2^ parameter and the quasi-likelihood under the independence model criterion.

**Results:** Data from 8,090 visits (550 patients) from ACT-RAY and 775 visits (534 patients) from PEARL were analyzed. The best performing models for HAQ were the HUPI (*R*^2^ = 0.351) and SDAI ones (*R*^2^ = 0.329). For serum IL-6 levels, the SDAI (*R*^2^ = 0.208) followed by the HUPI model (*R*^2^ = 0.205). For radiographic progression in ACT-RAY, the HUPI (*R*^2^ = 0.034) and the DAS28 models (*R*^2^ = 0.026) performed best whereas the DAS28 (*R*^2^ = 0.030) and HUPI models (*R*^2^ = 0.023) did so in PEARL.

**Conclusions:** HUPI outperformed other indices identifying changes in HAQ and radiographic progression and performed similarly to SDAI for IL-6 serum levels.

## Introduction

Routine management of rheumatoid arthritis (RA) using the treat-to-target (1) and tight-control (2) strategies require validated tools to measure disease activity. The most frequently used measures in randomized clinical trials (RCT) are the disease activity score of 28 joints (DAS28) (3), the simplified disease activity index (SDAI) (4), and the clinical disease activity index (CDAI) (5). Although extensively validated, these indices exhibit some limitations. Different cohorts have shown that DAS28 and SDAI may be sex-biased, as they include a pain rating and erythrosedimentation rate (ESR), both usually higher in women. This potential bias could lead rheumatologists to over-treat women with RA (6, 7).

To overcome these limitations, the “Hospital Universitario La Princesa Index” (HUPI), was developed and validated (8–10). HUPI includes the same variables as DAS28 but its calculation can be done either with ESR, C-Reactive Protein (CRP), or both, depending on their availability, as a way to tackle missing data (8). This index developed and validated in an early arthritis cohort (8), has disease activity cut-offs with higher areas under the curve in comparison to DAS28, SDAI, and CDAI (9). HUPI's responsiveness was evaluated against the other disease activity indices in three different scenarios, namely an RCT and two different RA cohorts, including patients with early and established disease. The responsiveness was similar to that of DAS28-CRP and better than the remaining indices with response criteria that are more stringent than those of EULAR (10). Based on its psychometric properties, the 2019 update of the American College of Rheumatology recommended RA disease activity measures included HUPI among the indices that fulfil minimum standards for regular use in most clinical settings (11).

Nowadays, the importance of an early diagnosis and treatment in patients with RA is well-established (12, 13). However, to offer patients a tailored therapy aimed at improving efficacy and reducing side effects, we need reliable measures of what is happening now (assessment) and what will happen in the future (prediction). Accordingly, we hypothesized that HUPI's performance to identify unbiased changes in disease activity makes it more suitable to assess changes in relevant outcomes and surrogates of inflammation (14, 15). The objective of this study was to compare the capacity of HUPI and other indices to identify changes in (i) physical function, (ii) serum levels of interleukin-6 (IL-6), and (iii) radiographic progression in patients with early and established RA.

## Methods

This study is a secondary data analysis of an early arthritis cohort and an RCT in established RA.

### Study Population

#### The ACT-Ray Trial

The main characteristics of the ACT-RAY trial have been previously reported (16). In summary, this is a 3-year double-blind RCT designed to evaluate the efficacy and safety of tocilizumab (TCZ) plus methotrexate vs. TCZ monotherapy in patients with established RA with inadequate response to methotrexate. The study included patients fulfilling the ACR 1987 criteria with a DAS28 > 4.4 and erosive disease. Data on demographics, disease activity variables, and laboratory data were collected every 4 weeks from baseline until the end of the study. Since there were no statistically significant differences in clinical response between arms, we included all patients' data regardless of the allocation group up to week 52 when, according to the protocol, patients in sustained remission discontinued treatment with TCZ (16).

#### The PEARL Cohort

This prospective cohort has been previously described (9). In summary, PEARL includes incident cases of early arthritis, with one or more swollen joints for less than a year. Patients are referred by their treating rheumatologist to an early arthritis clinic, in which patients undergo 5 visits (at baseline, 26, 52, 104, and 260 weeks) per protocol performed by the same two rheumatologists, which guarantees consistency in clinical examination, particularly joint counts.

Demographics, disease activity measures, and radiological data are routinely recorded in standardized forms. In addition, biological samples are systematically collected. Patients are treated according to their treating rheumatologist's criteria.

For the present study, we included patients either meeting the 1987 ACR criteria for RA (17) or classified as having UA (18) at the 24-month follow-up visit, from cohort inception (2000) until June 2019.

### Variables

Physical function: It was measured through the Health Assessment Questionnaire-Disability Index (HAQ) in both datasets. This self-reported questionnaire was administered at every follow-up visit using cross-cultural validated versions (19, 20).

#### Serum IL-6 Levels (pg/ml)

IL-6 had been previously measured in frozen serum samples from PEARL patients using an enzyme-linked immunoassay (Quantikine^®^HS ELISA, R&D Systems^®^) according to the manufacturer's instructions as previously described (21). The biobank of La Princesa University Hospital—Health Research Institute (ISS-IP) provided serum for this previous study. In the present work, we have used these previous serum IL-6 measurements as a surrogate for inflammation in the PEARL study, to analyze their relationship with the different indices studied.

IL-6 was measured as a surrogate for inflammation (14) only in the PEARL study, using an enzyme-linked immunoassay (Quantikine^®^HS ELISA, R&D Systems^®^) according to the manufacturer's instructions.

#### Radiographic Progression

Plain X-rays were available to measure radiographic progression using the Genant-Sharp score in ACT-RAY (22) and the modified-Sharp-Van der Heijde score (23) (applied only in hands) in PEARL. We analyzed only the Δ of erosions because we consider it more accurate to show changes only due to RA, as opposed to measuring changes in joint space narrowing that have been shown to be strongly associated with age, rather than disease activity (24). The variable Δ of erosions was calculated as the difference in the respective scores between baseline and the 52-week visit for ACT-RAY and the 104-week follow-up visit for PEARL.

Disease activity indices included DAS28-ESR, SDAI, and HUPI and were calculated as follows:

1. DAS28-ESR = 0.56^*^√(TJC28) + 0.28^*^√(SJC28) + 0.70^*^ ln(ESR) + 0.014^*^(GDAPat). TJC28 and SJC28 refer to the count of tender and swollen joints in 28 joints while GDAPat does so for the patients' global disease assessment (25).
2. SDAI = TJC28 + SJC28 + CRP + GDAPat + GDAPhy. The latter refers to the physicians' global disease assessment (5).
3. HUPI is calculated as the sum of four variables (graded 0–3, see Supplementary Table 1): TJC28, SJC28, GDAPhy, and acute phase reactants (the average score value of ESR and CRP must be used if both are considered (9).

Categories of disease activity were established based on published cut-offs (5, 8, 25, 26).

### Statistical Analysis

Data from each of the two studies were analyzed independently. Normally distributed variables were represented as mean and standard deviation (SD) and non-normally distributed variables as the median and interquartile range (IQR). Categorical variables were presented as numbers and proportions.

To assess the performance of HUPI, DAS28, and SDAI on explaining changes in the three mentioned outcomes, we developed models for each of them as dependent variable adjusting for known potential confounders, such as age and sex (27). Only patients without missing data in all of these variables were included for analysis. For all models, indices and age were standardized (centered and scaled by subtracting from each variable record the variable mean value and dividing the result by the standard deviation), thereby allowing comparisons.

Models with HAQ as a dependent variable were developed in ACT-RAY and fitted using Generalized Estimating Equations (GEE), nesting visits to each patient. An unstructured variance-covariance matrix for fixed and residual terms was used to avoid assumptions on the variance-covariance structure. Models were ranked according to the *R*^2^ parameter and the quasi-likelihood under the independence model criterion (QIC) (28). The model with the highest *R*^2^ and the lowest QIC was selected as the best-ranked one. This ranking was then validated in PEARL using the *R*^2^ parameter.

We used a similar approach to develop models for IL-6 serum levels as the dependent variable. As IL-6 levels were not collected in ACT-RAY, we used 80% of the PEARL population to establish the predicting model and the remaining 20% for its validation. This analysis was done with the R package “geepack” (29).

Finally, the models describing the relationship between Δ erosions and the different indices were developed independently for ACT-RAY and PEARL, because of the previously described differences in their measurement. For these models, we obtained the mean value of each disease activity index for the entire follow-up, rather than the score at every visit, as done in the previous models. These mean values were categorized as follows: remission = 0, low = 1, moderate = 2 and high activity = 3, according to their respective cut-offs (3). Models were ranked by the *R*^2^ parameter (R package stats) and the AIC (Akaike's Information Criterion) (30, 31), being the one with the highest *R*^2^ and the lowest AIC selected as the best-ranked model. The relative importance of each predictor was calculated by decomposing the *R*^2^ value of the model into components corresponding to each predictor (R package r2glmm) (32). Linear models were used to analyze HAQ and IL-6 and quadratic ones for radiographic progression due to better data adjustment. Statistical analyses were conducted using R version 3.6.3 (27).

### Ethical Considerations

This is a secondary analysis of anonymized data from patients included in the ACT-RAY and PEARL studies. The ACT-RAY trial was approved by the Ethics committees of each participant center (see Acknowledgement section “ACT-RAY group”) and the PEARL study was approved by the Ethics Committee for Clinical Research at the Hospital Universitario de La Princesa (PI-518; March 28th, 2011). All patients had signed a written consent form before inclusion. Both studies were conducted according to the principles of the Helsinki Declaration (33).

## Results

The analysis included 8,090 visits from 550 patients in ACT-RAY and 775 visits from 534 patients in PEARL. Nonetheless, different numbers of visits/patients were assessed for each model, based on the availability of data for the involved variables (see further details in each table). Patients' demographic and clinical characteristics are presented in Supplementary Table 2. In the complete sample (*n* = 1,084), 80% of patients were women, and 29% current smokers. Patients in the RCT presented higher HAQ and disease activity at baseline than their counterparts in the early arthritis cohort.

### Comparative Analysis of Indices With HAQ as Outcome

**Figure 1:**
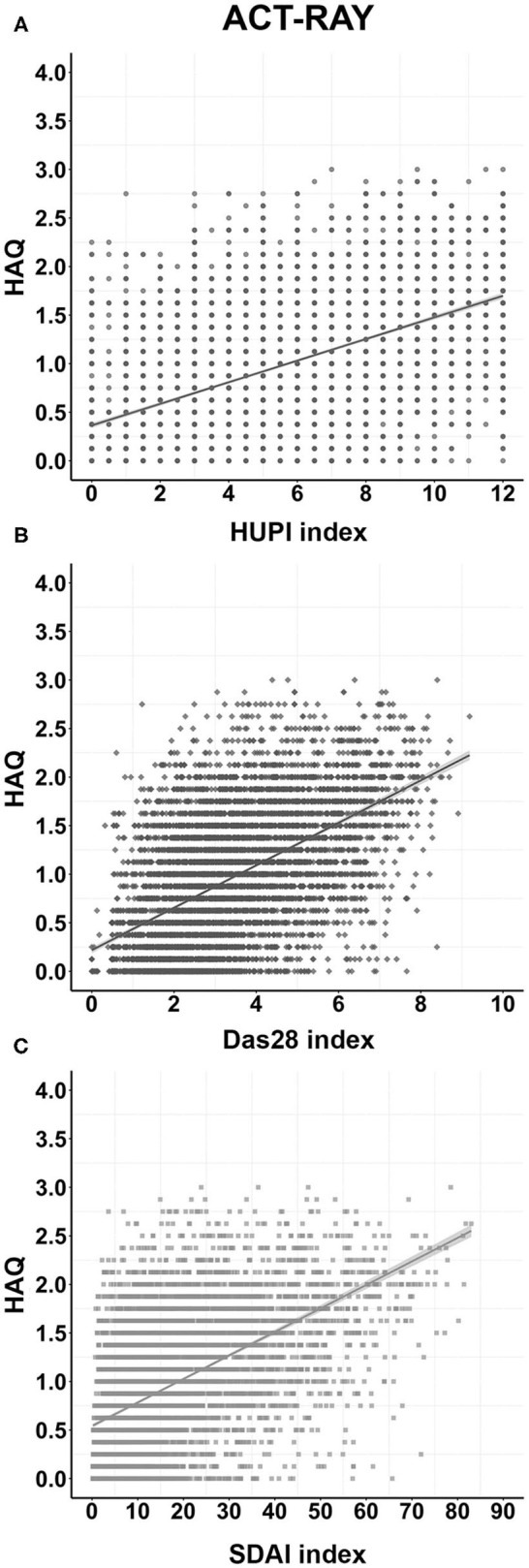
Distribution of HAQ according to each disease activity index in the ACT-RAY study through follow-up. **(A)**, **(B)**, and **(C):** Distributions according to the HUPI, DAS28, and SDAI index, respectively. Data are shown as dot-plots and their fitted linear prediction (line) with 95% confidence intervals (grey shadow).

The model fitted to explain HAQ adding HUPI as a predictor presented the highest *R*^2^ (0.351) and the lowest QIC (1989.790) compared to the models using SDAI (*R*^2^: 0.329; QIC: 2057.011) and DAS28 (*R*^2^: 0.325; QIC: 2070.581) indicating that the former explained 35% of the HAQ variance, while the latter two indices explained ~33%. In the same line, the β coefficient for HUPI was 0.365 vs. 0.344 and 0.343 for DAS28 and SDAI, respectively. The *R*^2^ parameters of all models remained similar in the validation cohort. Other parameters of each model are shown in Table 1 and the distribution of HAQ according to HUPI, DAS28, and SDAI in both study populations are shown in Figure 1 and Supplementary Figure 1.

**Table 1:** Models for HAQ comparing the performance of different indices.

**Model**	**Predictors**	**β**	**SE**	***P***	**QIC**	**$R_{\text{model}}^{2}$**	$R_{\text{validation}}^{2}$^*^
HUPI	Intercept	0.676	0.014	<10^−4^	1989.790	0.351	0.417
	Sex	0.335	0.016	<10^−4^			
	Age	0.098	0.007	<10^−4^			
	HUPI	0.365	0.006	<10^−4^			
DAS28	Intercept	0.744	0.014	<10^−4^	2070.581	0.325	0.440
	Sex	0.253	0.016	<10^−4^			
	Age	0.096	0.007	<10^−4^			
	DAS28	0.344	0.007	<10^−4^			
SDAI	Intercept	0.694	0.014	<10^−4^	2057.011	0.329	0.420
	Sex	0.314	0.016	<10^−4^			
	Age	0.099	0.007	<10^−4^			
	SDAI	0.343	0.007	<10^−4^			

*Data from the variables age, HUPI, DAS28, and SDAI were scaled and centered. β, β-coefficients; SE, Standard Error; QIC, quasi-likelihood under the Independence model criterion; HAQ, Health Assessment Questionnaire; HUPI, Hospital Universitario La Princesa Index; DAS28, Disease Activity Score of 28 joints; SDAI, Simplified Disease Activity Index*.

* *This model was developed with data from ACT-RAY (541 patients and 6778 visits) and validated in PEARL. (532 patients and 2032 visits)*.

### Comparative Analysis of Indices With IL-6 Serum Levels as Outcome

**Figure 2:**
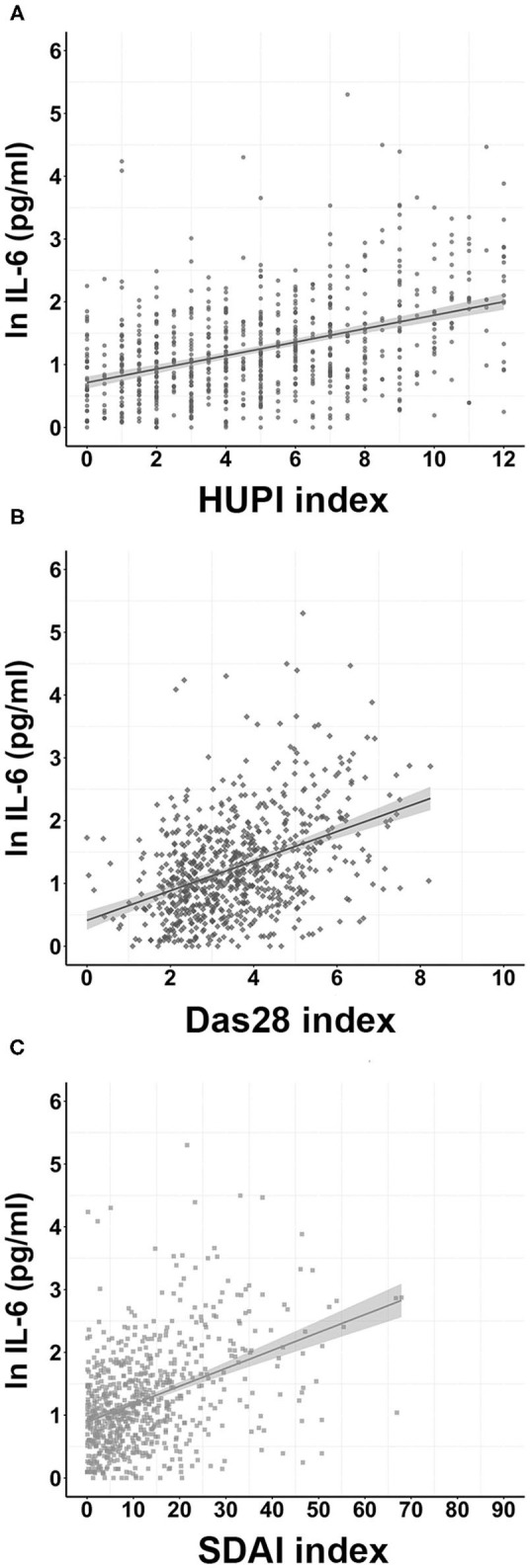
Distribution of IL-6 serum levels according to each disease activity index in the PEARL study through follow-up. **(A)**, **(B)**, and **(C)**: Distributions according to the HUPI, DAS28, and SDAI index, respectively. IL-6 level values were transformed to their natural logarithm (nl). Data are shown as dot-plots and their fitted linear prediction (line) with 95% confidence intervals (grey shadow).

Variations in the IL-6 serum level were initially modeled with a randomly split 80% of the PEARL population. In these initial models, IL-6 levels were better explained when including SDAI or HUPI as predictors, with *R*^2^ of 0.208 (QIC: 289.207) and 0.205 (QIC: 290.823), respectively, in comparison with an *R*^2^ of 0.190; QIC: 295.610 for DAS28 (Table 2). These results indicate that the former two explained ~21% of the variance, while the latter explained 19%. The β coefficient for SDAI was 0.363 vs. 0.345 and 0.337 for HUPI and DAS28, respectively. Of note, the *R*^2^ parameters of HUPI and DAS28 remained similar when applied to the validation cohort (the remaining 20% of the PEARL population), as opposed to the SDAI model, which changed from explaining ~21% in the initial population to 18% in the validation population (Table 2). It is also noteworthy that sex only reached significance in the DAS28 model. Additional data of the models are presented in Table 2 and the distribution of IL-6 serum levels according to each index scale in Figure 2.

**Table 2:** Models for IL-6 levels comparing the performance of different indices.

**Model**	**Predictors**	**β**	**SE**	***P***	**QIC**	**$R_{\text{model}}^{2}$**	$R_{\text{validation}}^{2}$^*^
HUPI	Intercept	1.034	0.140	<10^−4^	290.823	0.205	0.210
	Sex	−0.117	0.070	0.093			
	Age	0.006	0.002	0.003			
	HUPI	0.345	0.033	<10^−4^			
DAS28	Intercept	1.129	0.140	<10^−4^	295.610	0.190	0.208
	Sex	−0.224	0.071	0.002			
	Age	0.005	0.002	0.005			
	DAS28	0.337	0.034	<10^−4^			
SDAI	Intercept	0.997	0.135	<10^−4^	289.207	0.208	0.176
	Sex	−0.119	0.068	0.083			
	Age	0.007	0.002	<10^−3^			
	SDAI	0.363	0.035	<10^−4^			

*Data from the variables HUPI, DAS28, and SDAI were scaled and centered. β, β-coefficients; SE, Standard Error; QIC, quasi-likelihood under the Independence model criterion; IL-6, Interleukin-6; HUPI, Hospital Universitario La Princesa Index; DAS28, Disease Activity Score of 28 joints; SDAI, Simplified Disease Activity Index.*

* *This model was developed with 80% of data from PEARL (201 patients and 543 visits) and validated in the remaining 20%. (111 patients and 141 visits)*.

### Comparative Analysis of Indices With Radiographic Progression as Outcome

As radiographic progression was evaluated using different methodologies in both studies, we ran separate comparative analyses. As shown in Table 3, when analyzing data from the ACT-RAY study, the model including HUPI as an explanatory variable showed the best performance (*R*^2^: 0.034; AIC:925.687), followed by the one with DAS28 (*R*^2^: 0.026; AIC: 928.793) and then the one using SDAI (*R*^2^: 0.017; AIC:932.347). These results indicate that the HUPI model explained slightly better the variance of Δ erosions (3.4%) than the models including DAS28 and SDAI (2.6 and 1.7%, respectively). When assessing partial *R*^2^ parameters for each explanatory variable, HUPI and HUPI^2^ explained 2% of the variance, while DAS28/DAS28^2^ explained 1% and SDAI/SDAI^2^ 0.2%. β coefficients for HUPI and HUPI^2^ were 1.472 and 1.632 vs. 1.675 and 0.266 for DAS28/DAS28^2^ and 0.759 and −0.042 for SDAI/SDAI^2^, respectively. Additional data are shown in Table 3.

**Table 3:** Models for radiographic progression in the ACT-RAY study comparing the performance of different indices.

**Model**	**Predictors**	**β**	**SE**	***P***	**AIC**	$R_{\text{model}}^{2}$^*^
HUPI	Intercept	0.189	0.097	0.051	925.687	0.034
	Sex	−0.260	0.107	0.016		
	Age	−0.021	0.041	0.608		
	HUPI	1.472	0.801	0.066		
	HUPI^2^	1.632	0.804	0.043		
DAS28	Intercept	0.206	0.098	0.036	928.793	0.026
	Sex	−0.280	0.108	0.100		
	Age	−0.029	0.041	0.483		
	DAS28	1.675	0.808	0.038		
	DAS28^2^	0.266	0.808	0.741		
SDAI	Intercept	0.192	0.098	0.051	932.347	0.017
	Sex	−0.263	0.108	0.015		
	Age	−0.028	0.041	0.495		
	SDAI	0.759	0.809	0.348		
	SDAI^2^	−0.042	0.809	0.958		

*Data from the variables age, HUPI, DAS28, and SDAI were scaled and centered. β, β-coefficients; SE, Standard Error; AIC, Akaike's Information Criterion; HUPI, Hospital Universitario La Princesa Index; DAS28, Disease Activity Score of 28 joints; SDAI, Simplified Disease Activity Index*.

* *This model was developed with data from ACT-RAY (550 patients)*.

**Figure 3:**
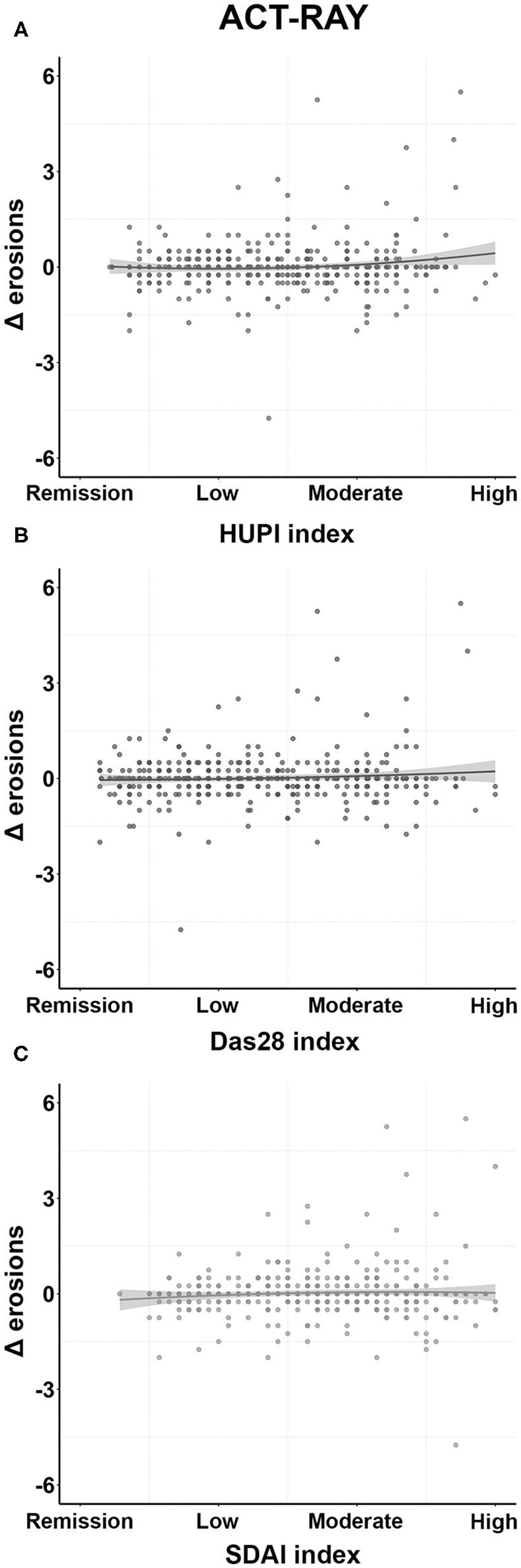
Distribution of Δ erosions according to the different disease activity indices in the ACT-RAY study. **(A)**, **(B)**, and **(C)**: Distributions according to HUPI, DAS28, and SDAI categories, respectively. Disease activity values represent patients' mean disease activity through follow-up. Data are shown as dot-plots and their fitted linear prediction (line) with 95% confidence intervals (grey shadow).

In contrast, when using data from PEARL, none of the models were associated with radiographic progression. Results were *R*^2^: 0.030 (0.010–0.150) AIC: 347.520) for DAS28, *R*^2^: 0.023 (0.008–0.138) AIC: 348.413 for HUPI, and *R*^2^: 0.018 (0.007–0.131) AIC: 348.955 for SDAI. The model including DAS28 explained ~3% of the variance, while those with HUPI and SDAI explained 2.3% and 1.8%, respectively. Partial *R*^2^ parameters show that DAS28/DAS28^2^ explained 2.5% of the overall variance, while HUPI and SDAI explained 1.7 and 1.3%, respectively. Additional details are shown in Supplementary Table 3. The distribution of the variable Δ erosions in ACT-RAY and PEARL according to the different categories of each index is shown in Figure 3 and Supplementary Figure 2, respectively.

## Discussion

In this study, we evaluated the performance of HUPI in comparison to other traditional disease activity indices as explanatory variables for physical function decline measured by HAQ, inflammation, assessed by IL-6 serum levels, and radiographic progression measured by Δ erosions. Our results indicate that HUPI performed well with most outcomes studied, being the best in explaining the decline in physical function and radiographic progression (ACT-RAY) and second-best for IL-6 serum levels. Of note, all indices performed poorly with regard to radiographic progression, mainly because both populations showed modest changes in their respective radiographic scores, as expected for early diagnosed, intensively treated patients.

Even though the models containing HUPI did not outperform their counterparts in all comparisons, they were the most consistent in the different proposed scenarios. The SDAI models performed best for IL-6 changes, probably because the weight of CRP is high in SDAI but were the last ranked for Δ erosions. Similarly, DAS28 models worked best for explaining Δ erosions in PEARL but rated the worst for HAQ and IL-6.

The association between HAQ and traditional indices (DAS28, SDAI, and CDAI) has been previously analyzed in a study by Aletaha et al. (34) with two observational cohorts, one including patients with established RA, and another with early arthritis. These analyses showed moderate and similar correlations for all indices (*r* = 0.45–0.47) for the former, and weaker for the latter cohort (*r* = 0.26–0.31). Another study pooling data from three RCTs showed moderate to good correlations with HAQ for SDAI and CDAI at baseline and after 6 months of follow-up (*r* = 0.36–0.66) (4). These observations are consistent with our results: SDAI and DAS28 performed quite similarly on HAQ assessment. Nonetheless, our data support a slight superiority of HUPI.

The association between indices and IL-6 levels has also been previously analyzed in a study by Madhok et al. (35) showing a weak correlation (*r* = 0.3) with the Ritchie Activity Index. In our study, initial models including all three indices performed similarly, with little differences favoring those including SDAI (with *R*^2^ parameters ranging from 0.190 to 0.208). Notably, when validating these models with the 20% remaining data from PEARL, HUPI and DAS28 models performed better than SDAI.

Navarro-Compán et al. (36) summarized the association between disease activity indices and radiographic progression in a systematic review. The majority of studies reported a significant association, especially after adjustment by time. However, this review did not carry out comparative analyses between indices.

Aletaha et al. (34) assessed the linear correlation between time-averaged DAS28, SDAI, and CDAI and radiographic progression (measured with the Larsen score) after 3 years of follow-up, and found similar moderate correlations, with r coefficients ranging from 0.54 to 0.59. Of note, in this study, no GEE modeling was carried out. Klarenbeek et al. (37) using 5-year data from the BeST study, found similar results after assessing the association of different indices with radiographic progression, using the Sharp-van der Heijde score, and HAQ. These authors ran GEE models to analyze different scenarios for both outcomes and found that all associations were highly comparable. Despite the limited radiographic progression in ACT-RAY and PEARL, our results are in line with those previously described in the literature, favoring HUPI's performance to explain radiographic progression.

Our study has strengths, such as a study population including patients with both early and established RA, as well as a thorough statistical analysis. Nonetheless, it also presents some limitations, the most important being the low radiographic progression observed in both cohorts, which might have affected the performance of the three disease activity indices. This prevented us from establishing firm conclusions from the comparative analysis. Another limitation is the fact that IL-6 serum levels were only available from PEARL, something that limited the number of visits/patients assessed.

In conclusion, HUPI exhibits a slightly superior performance to identify physical function declines and radiographic progression than DAS28 and SDAI and detects changes in IL-6 serum levels similar to the other indices. This behavior is consistent in early and established RA. These new findings, in addition to the absence of sex bias and the possibility of its calculation either with CRP or ESR, reinforce the role of HUPI for research purposes.

## Data Availability Statement

The data analyzed in this study is subject to the following licenses/restrictions: Data from the PEARL study can be requested to the corresponding author. Data from ACT-RAY were provided by Hoffmann-La Roche Ltd through a data-sharing agreement that does not allow for the public sharing of these data. The authors did not enjoy any special access privileges in gaining access to these data. Regarding the possibility that any other researcher would like to request data to replicate the reported study findings, Hoffmann-La Roche Ltd has implemented a Data Sharing policy to align with the ICMJE recommendations: “Qualified researchers may request access to individual patient-level data through the clinical study data request platform (www.clinicalstudydatarequest.com). Further details on Roche's criteria for eligible studies are available in https://clinicalstudydatarequest.com/Study-Sponsors/Study-Sponsors-Roche.aspx. For further details on Roche's Global Policy on the Sharing of Clinical Information and how to request access to related clinical study documents, see https://www.roche.com/research_and_development/who_we_are_how_we_work/clinical_trials/our_commitment_to_data_sharing.html”. Requests to access these datasets should be directed to ACT-RAY data: www.clinicalstudydatarequest.com. PEARL data: Isidoro Gonzalez-Alvaro, isidoro.ga@ser.

## Ethics Statement

This is a secondary analysis of anonymized data from patients included in the ACT-RAY and PEARL studies. The ACT-RAY trial was approved by the Ethics committees of each participant center and the PEARL study was approved by the Ethics Committee for Clinical Research at the Hospital Universitario de La Princesa (PI-518; March 28th, 2011). Both studies were conducted according to the principles of the Helsinki Declaration. The patients/participants provided their written informed consent to participate.

## Author Contributions

IG-Á, SR-G, and NM contributed to conception and design of the study and organized the database. NM performed the statistical analysis and wrote a section of the manuscript. SR-G wrote the first draft of the manuscript. All authors contributed to manuscript revision, read, and approved the submitted version.

## The ACT-RAY Study Group

Lead authors: Dougados, M. CHU Paris Centre—Hôpital Cochin. France. E-mail: maxime.dougados@cch.ap-hop-paris.fr; Huizinga, T. Leiden University Medical Center, Netherlands. E-mail: T.W.J.Huizinga@lumc.nl

Abu Shakra, M. Soroka Medical Center. Israel

Alberts, A. West Broward Rheumatology Associates, Inc. United States

Alperi Lopez, M. Hospital Univ. Central de Asturias. Spain

Amital, H. Chaim Sheba Medical Center. Israel

Aringer, M. Universitatsklinikum “Carl Gustav Carus”. Germany

Aslanidis, S. Hippokratio Hospital. Greece

Berenbaum, F. Hôpital Saint Antoine. France

Bijlsma, H. Academisch Medisch Centrum Utrecht. Netherlands

Blanco-Garcia, FJ. Complejo Hospitalario Universitario A Coruña. Spain

Bliddal, H. Frederiksberg Sygehus. Denmark

Borofsky, M. Clinical Research Center of Reading. United States

Brocq, O. Ch Princesse Grace. Monaco

Buldakov, S. Republican Clinicodiagnostic Center. Russian Federation

Cantini, F. Presidio Ospedaliero Misericordia e Dolce. Italy

Carreño-Perez, L. Hospital General Universitario Gregorio Marañon. Spain Chahade, W. Hospital Estadual do Servidor Publico. Brazil

Ciconelli, R. Universidade Federal de São Paulo. Brazil

Codreanu, C. Centrul de Boli Reumatismale Dr. Ioan Stoia. Romania

Dahlqvist, SR. Norrlands Universitary Hospital. Sweden

Damjanov, N. Institut Za Reumatologiju. Serbia

Diamantopoulos, A. Sørlandet Sykehus Kristiansand. Norway

Dimdina, L. Clinical University Hospital Gailezers. Latvia

Dimic, A. Institut Za Prevenciju, Lecenje I Rehabilitaciju. Serbia

Dorokhov, A. State Institution of Health Care—Territorial Clinical Hospital. Russian Federation

Dubikov, A. City Clinical Hospital # 2. Russian Federation

Fadienko, G. Glpu Tjumen Regional Clinical hospital #1. Russian Federation

Fanø, N. Sjællands Universitetshospital, Køge. Denmark

Ferreira, G. Hospital das Clinicas–UFMG. Brazil

Gabrielli, A. Uni Politecnica Delle Marche; Ist. Di Clinica Medica Generale Ematologia Ed Immunologia Clinica. Italy

Gaffney, K. Norfolk & Norwich Hospital. United Kingdom

Gaudin, P. Hopital Sud. France

Gerlag, DM. Academisch Medisch Centrum. Netherlands

Gerli, R. Osp S. Maria Misericordia Dip. Italy

Gonçalves, CR. Hospital das Clínicas–FMUSP. Brazil

Hansen, MS. Gentofte Hospital. Denmark

Hanvivadhanakul, P. Thammasat University Hospital. Thailand

Høili, C. Sykehuset Ostfold Moss HF. Norway

Hou, A. Inland Rheumatology; Clinical Trials, Inc. United States

Hunter, J. Gartnavel General Hospital. United Kingdom

Ilic, T. Clinical Centre of Vojvodina. Serbia

Ionescu, R. Spitalul Sf Maria. Romania

Kaine, J. Sarasota Arthritis Center. United States

Kakurina, N. Clinical Hospital of Daugavpils. Latvia

Kamalova, R. Republican clinical hospital. Russian Federation

Kelly, T. Innovative Health Research. United States

Knyazeva, L. GMU Kursk Regional Clinical Hospital. Russian Krumina Federation, L. L.Krumina GP practice. Latvia

Kurthen, R. Praxis Dr. med. Reiner Kurthen. Germany

Lagrone, RP. St. Thomas Hospital. United States

Lapadula, G. Ospedale Policlinico Di.M.I.M.P. Italy

Lavrentjevs, V. P.Stradins Clinical University Hospital. Latvia

Lawson, JG. Piedmont Arthritis Clinic. United States

Lazic, Z. Clinical Center Kragujevac. Serbia

Lejnieks, A. Rakus Clinic Linezers. Latvia

Levy, Y. Meir Medical Center. Israel

Lexberg, Å. Drammen sykehus Vestre Viken HF. Norway

Mader, R. Haemek Hospital. Israel

Mariette, X. Ch De Bicêtre. France

Markovits, D. Rambam Medical Center. Israel

Martin Mola, E. Htal. La Paz. Spain

Maugars, Y. Hopital Hotel Dieu Et Hme. France

Maymo Guarch, J. Hospital del Mar. Spain

Mazurov, VI. Sbei Of Hpe “Northwestern State Medical University N.A. I.I.Mechnikov”. Russian Federation

Mikkelsen, K. Revmatismesykehuset. Norway

Morovic Vergles, J. Clinical Hospital Dubrava. Croatia

Nabizadeh, S. Martina Hansen Hospital. Norway

Nanagara, R. Khon Kaen University. Thailand

Nasonov, EL. Fsbi “Scientific Research Institute of Rheumatology” Of Russian Academy Of Medical Sciences. Russian Federation

Navarro-Sarabia, F. Hospital Universitario Virgen Macarena. Spain

Neumann, T. Universitatsklinikum Jena. Germany

Novak, S. Rheumatology and Clinical Immunology. Croatia

Olech, E. Oklahoma Medical Research Foundation. United States

Oza, M. Arthritis/Osteoporosis Treatment Center. United States

Paran, D. Sourasky / Ichilov Hospital. Israel

Parsik, E. North Estonian Regional Hospital. Estonia

Pegram, S. Rheumatic Disease Clin Res Ctr. United States

Pombo-Suarez, M. Hospital Nuestra Señora de la Esperanza. Spain

Popova, T. Municipal Autonomous Institution of Healthcare “City Clinical Hospital #40”. Russian Federation

Puechal, X. Ch Du Mans. France

Raja, N. Agilence Arthritis and Osteoporosis Medical Center, Inc. United States

Ridley, D. St. Paul Rheumatology. United States

Rosner, I. Bnei Zion Medical Center. Israel

Rubbert-Roth, A. Klinik der Uni zu Ko ln. Germany

Rudin, A. Sahlgrenska Universitetssjukhuset. Sweden

Saraux, A. Hôpital La Cavale Blanche. France

Saulite-Kandevica, D. D.Saulite-Kandevica Private Practice. Latvia

Settas, L. Ahepa Hospital. Greece

Sfikakis, P. Laiko General Hospital. Greece

Sheeran, T. Cannock Chase Hospital. United Kingdom

Sizikov, A. FSBI Scientific Research Institute of Clinical Immunology of SB of RAMS. Russian Federation

Stamenkovic, D. Clinical Hospital Centre Rijeka. Croatia Stefanovic, D. Military Medical Academy. Serbia

Stolow, JB. Texas Arthritis Research Center. United States

Tan, AL. Chapel Allerton Hospital. United Kingdom

Tebib, J. Ch Lyon Sud. France

Tishler, M. Assaf Harofe. Israel

Tony, HP. Universitatsklinikum Würzburg. Germany

Troum, OM. United States

Uaratanawong, S. Vajira Hospital. Thailand

Ucar Angulo, E. Hospital de Basurto. Spain

Valenzuela, G. Berma Research Group. United States

van der Laken, K. VU Medisch Centrum. Netherlands

Van Laar, J. School of Clinical Medical Services. United Kingdom

Van RIEL, P.L.C.M. Akademisch Ziekenhuis St. Radboud. Netherlands Vasilopoulos, D. Hippocrateio Hospital of Athens. Greece

Veldi, T. East Tallinn Central Hospital. Estonia

Vinogradova, I. State Institution of Healthcare Ulyanovsk Regional Clinical Hospital. Russian Federation

Vosse, D. Academisch Ziekenhuis Maastricht. Netherlands

Wassenberg, S. Evangelisches Fachkrankenhaus. Germany

Weidmann, C. Medvin Clinical Research. United States

Weitz, M. Center For Arthritis. United States

Wollenhaupt, J. Scho n Klinik Hamburg-Eilbek Klinik für Rheumatologie. Germany Xavier, R. Hospital das Clinicas–UFRGS. Brazil

Yakupova, S. Kazan State Medical University. Russian Federation

Zagar, I. Klinicki Bolnicki Centar Zagreb. Croatia

Zavgorodnaja, T. P.Stradins Clinical University Hospital. Latvia

Zemerova, E. Khanty-Mansiysk Autonomous Area—Ugri Region Clinical Hospital. Russian Federation

Zisman, D. Carmel Hospital. Israel

Zonova, E. FSBI Scientific Research Institute of Clinical and Experimental Lymphology of SB of RAMS. Russian Federa.

## Conflict of Interest

The authors declare that the research was conducted in the absence of any commercial or financial relationships that could be construed as a potential conflict of interest.
