# Supplementary material for: Disease Activity Indices in Rheumatoid Arthritis: Comparative Performance to Detect Changes in Function, IL-6 Levels, and Radiographic Progression
Source: Front Med (Lausanne). 2021 May 31;8:669688. doi: 10.3389/fmed.2021.669688 (PMC8200542; doi:10.3389/fmed.2021.669688)
Supplement: Supplementary file 1 [file Data_Sheet_1.docx]

**SUPPLEMENTARY MATERIAL**


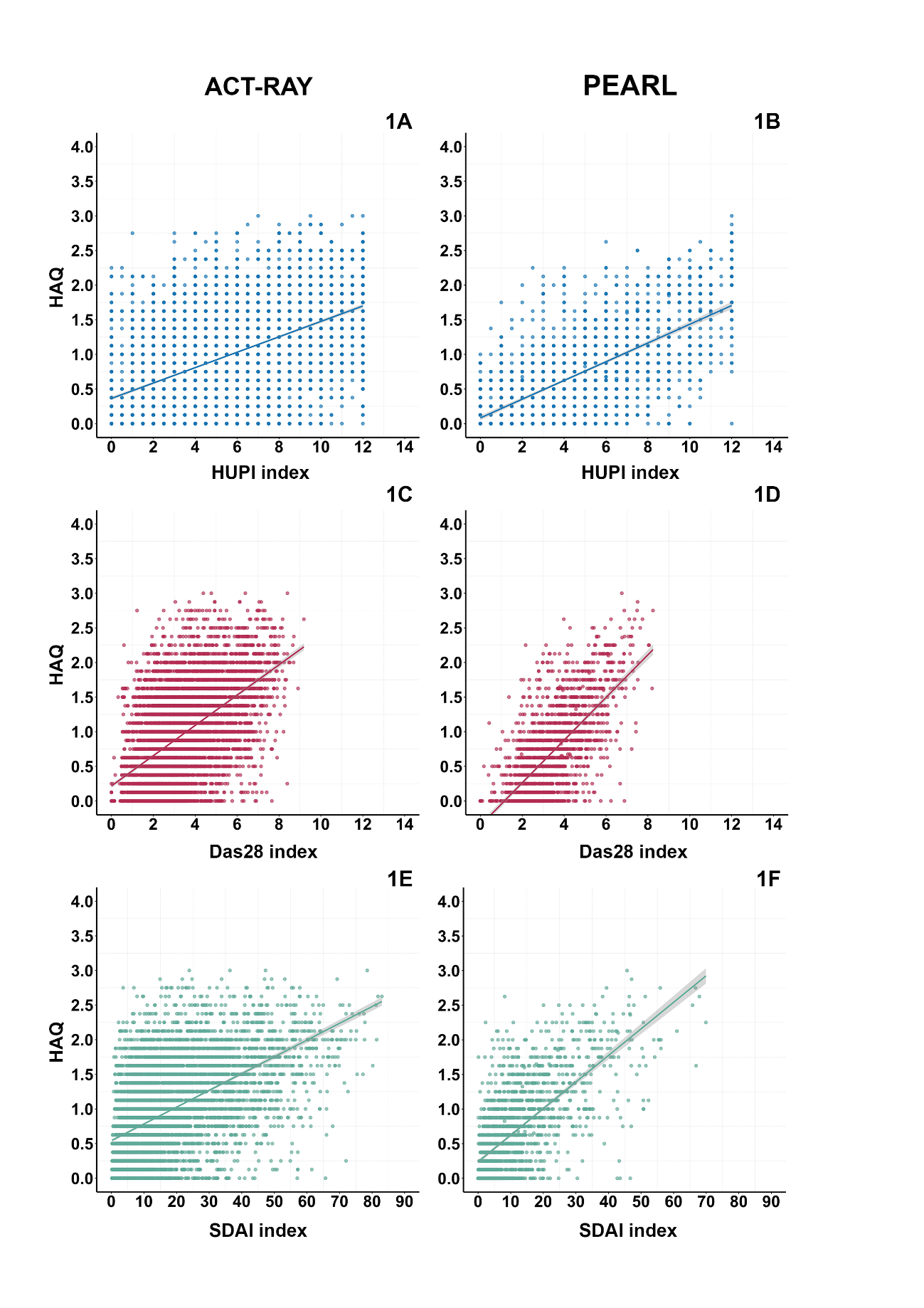


**Supplementary Figure 1.** Distribution of HAQ according to each disease activity index through follow-up. A and B. Distributions according to the HUPI index in patients from the ACT-RAY and PEARL studies, respectively. C and D. Distributions according to DAS28 in ACT-RAY and PEARL. E and F. Distributions according to SDAI in ACT-RAY and PEARL. Data are shown as dot-plots and their fitted linear prediction (line) with 95% confidence intervals (grey shadow).


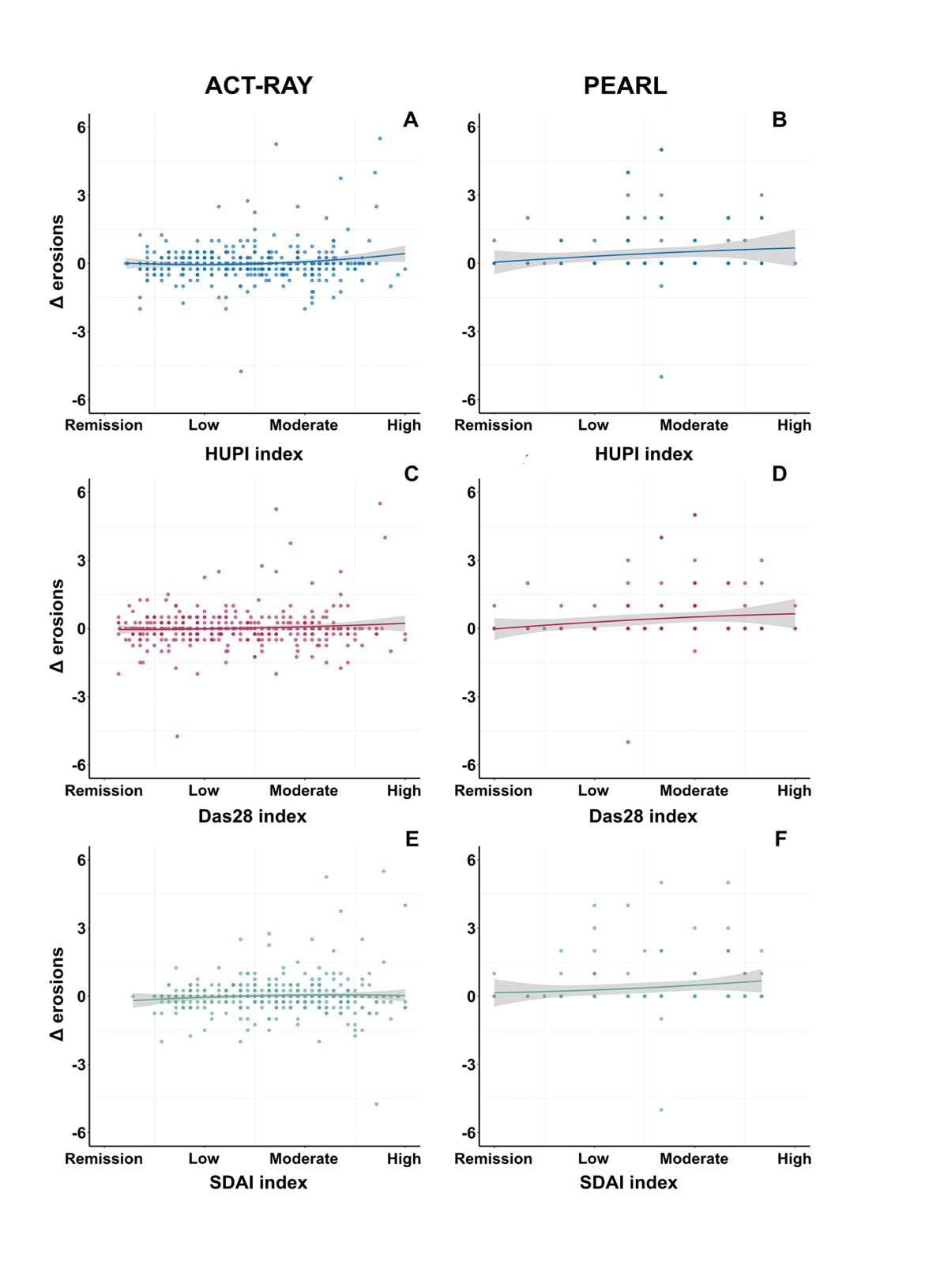


**Supplementary figure 2.** Distribution of Δ erosions according to each disease activity index in the ACT-RAY and PEARL studies. A and B: Distributions according to HUPI categories. C and D: Distributions according to DAS28 categories. E and F: Distributions according to SDAI categories. Disease activity values represent patients’ mean disease activity through follow-up. Data are shown as dot-plots and their fitted linear prediction (line) with 95% confidence intervals (grey shadow).

**Supplementary Table 1**. Scoring of the variables used to calculate HUPI.

|  |  | 0 | 1 | 2 | 3 |
| --- | --- | --- | --- | --- | --- |
| TJC28 | Female | 0 | 1-2 | 3-6 | >6 |
|  | Male | 0 | 1 | 2-3 | >3 |
| SJC28 |  | 0 | 1-2 | 3-4 | >4 |
| GDA-Patient (0-100) |  | 0-15 | 16-30 | 31-50 | >50 |
| C-reactive protein (mg/dl) |  | ≤0.1 | 0.11-0.8 | 0.81-1.5 | >1.5 |
| Erythrosedimentation rate | Female | 0-15 | 16-20 | 21-30 | >30 |
|  | Male | 0-10 | 11-15 | 16-20 | >20 |

TJC28: Tender joint count in 28 joints; SJC28: Swollen joint count in 28 joints; GDA-Patient: Patients’ global disease assessment.

**Supplementary Table 2**. Baseline demographic and clinical characteristics of the study population by study.

|  | ACT-RAY (n = 550) | PEARL (n=534) |
| --- | --- | --- |
| Female sex, n (%) | 443 (81) | 424 (79) |
| Age, years | 53.33 (12.67) | 55.91 (15.8) |
| Disease duration, years | 8.30 (8.20) | 0.55 (0.42) |
| Current smoker, n (%) | 196 (36) | 117 (23) |
| RF positive, n (%) | n.a. | 288 (54) |
| ACPA positive, n (%) | n.a. | 265 (50) |
| HAQ | 1.45 (0.62) | 1.04 (0.73) |
| IL-6 levels, (pg/ml) | n.a. | 8.18 (17.67) |
| SHS | n.a. | 3.86 (6.97) |
| GSS | 34.11 (36.83) | n.a. |
| HUPI | 10.96 (1.26) | 6.92 (3.30) |
| DAS28 | 6.33 (0.98) | 4.39 (1.52) |
| SDAI | 45.09 (13.74) | 20.30 (14.13) |

All categorical variables are expressed as number (%) and continuous variables as mean and standard deviation (SD). PEARL: Princesa Early Arthritis Longitudinal Study; RF: Rheumatoid Factor; ACPA: Anti-citrullinated peptide antibodies; HAQ: Health Assessment Questionnaire; IL-6: Interleukin 6; SHS: Sharp/Van der Heijde score; GSS: Genant/Sharp score; HUPI: Hospital Universitario La Princesa Index; n.a.: not applicable; DAS28: Disease Activity Score of 28 joints; SDAI: Simplified Disease Activity Index.

**Supplementary Table 3**. Models for radiographic progression in the PEARL study comparing the performance of different indices.

| Models | Predictors | β | SE | *P* | AIC | R^2^_model_ |
| --- | --- | --- | --- | --- | --- | --- |
| HUPI | Intercept | 0.399 | 0.188 | 0.036 | 348.413 | 0.023 |
|  | Sex | -0.085 | 0.220 | 0.697 |  |  |
|  | Age ^d^ | 0.046 | 0.099 | 0.640 |  |  |
|  | HUPI | 1.462 | 1.080 | 0.178 |  |  |
|  | HUPI^2^ | 0.404 | 1.042 | 0.698 |  |  |
| DAS28 | Intercept | 0.451 | 0.192 | 0.021 | 347.520 | 0.030 |
|  | Sex | -0.156 | 0.227 | 0.495 |  |  |
|  | Age | 0.038 | 0.099 | 0.702 |  |  |
|  | DAS28 | 1.880 | 1.116 | 0.095 |  |  |
|  | DAS28^2^ | 0.131 | 1.037 | 0.900 |  |  |
| SDAI | Intercept | 0.393 | 0.188 | 0.038 | 348.955 | 0.018 |
|  | Sex | -0.078 | 0.200 | 0.722 |  |  |
|  | Age | 0.055 | 0.099 | 0.574 |  |  |
|  | SDAI | 1.243 | 1.075 | 0.250 |  |  |
|  | SDAI^2^ | 0.355 | 1.044 | 0.734 |  |  |

Data from the variables age, HUPI, DAS28 and SDAI were scaled and centered. β: β-coefficients; SE: Standard Error; AIC: Akaike's Information Criterion; HUPI: Hospital Universitario La Princesa Index; DAS28: Disease Activity Score of 28 joints; SDAI: Simplified Disease Activity Index.

**This model was developed with data from PEARL. (150 patients)*
